# circEPSTI1 regulates ovarian cancer progression via decoying miR‐942

**DOI:** 10.1111/jcmm.14260

**Published:** 2019-03-19

**Authors:** Jing Xie, Shufen Wang, Genlin Li, Xia Zhao, Feng Jiang, Jie Liu, Weige Tan

**Affiliations:** ^1^ Department of Gynaecology and Obstetrics The First Affiliated Hospital of University of South China Hengyang Hunan China; ^2^ Breast Surgery Department The First Affiliated Hospital of Guangzhou Medical University, Guangzhou Medical University Guangzhou China

**Keywords:** circEPSTI1, circular RNAs, competitive endogenous RNAs, EPSTI1, miR‐942, ovarian cancer

## Abstract

Increasing studies show that circular RNAs (circRNAs) play vital roles in tumour progression. But, how circRNAs function in ovarian cancer is mostly unclear. Here, we detected the expression of circEPSTI1 in ovarian cancer and explored the function of circEPSTI1 in ovarian cancer via a series of experiments. Then, we performed luciferase assay and RNA immunoprecipitation (RIP) assay to explore the competing endogenous RNA (ceRNA) function of circEPSTI1 in ovarian cancer. qRT‐PCR verified that circEPSTI1 was overexpressed in ovarian cancer. Inhibition of circEPSTI1 suppressed ovarian cancer cell proliferation, invasion but promoted cell apoptosis. Luciferase assays and RIP assay showed that circEPSTI1 and EPSTI1 (epithelial stromal interaction 1) could directly bind to miR‐942. And circEPSTI1 could regulate EPSTI1 expression via sponging miR‐942. In summary, circEPSTI1 regulated EPSTI1 expression and ovarian cancer progression by sponging miR‐942. circEPSTI1 could be used as a biomarker and therapeutic target in ovarian cancer.

## 1 INTRODUCTION

Ovarian cancer is one of the major gynaecologic malignancies, with 295 414 new cases and 184 799 death in 2018 worldwide.^1^ Although most patients respond to the first treatment, the prognosis is still poor. Thus, it is urgent to explore molecular mechanisms of ovarian cancer progression to develop novel therapeutic targets for ovarian cancer.

Circular RNAs (circRNAs) have been implicated closely associated with cancer development.^2^ In esophageal carcinoma, circ0043898 acts as a tumour inhibitor that inhibits cell proliferation, migration and invasion and induces cell apoptosis and death.^3^ In non‐small cell lung cancer, circRNA F‐circEA‐2a acts as a tumour promoting circRNA that promotes cell migration and invasion.^4^ But the functions of most circRNAs remain unknown in ovarian cancer.

circRNAs are reported to regulate gene expression as miRNA sponges or competitive endogenous RNAs (ceRNAs).^5^ In breast cancer, circZNF609 promotes cell growth, migration and invasion via sponging miR‐145‐5p.^6^ In bladder cancer, circRNA BCRC‐3 functions as a tumour inhibitor to suppress cell proliferation by sponging miR‐182‐5p.^7^ In triple‐negative breast cancer, circEPSTI1 sponges miR‐4753 and miR‐6809 to regulate BCL11A expression and affect cell proliferation and apoptosis.^8^ These findings indicate that circRNAs could regulate cancer progression via ceRNA mechanism.

Here, we detected the expression of circEPSTI1 in 50 paired ovarian cancer tissue and adjacent normal tissues. We found that circEPSTI1 was up‐regulated in ovarian cancer. Inhibition of circEPSTI1 suppressed cell proliferation and invasion, but induced apoptosis. Moreover, circEPSTI1 could bind to miR‐942 and regulate the expression of EPSTI1 (epithelial stromal interaction 1). Therefore, circEPSTI1 could be used as a biomarker and therapeutic target for ovarian cancer.

## 2 MATERIALS AND METHODS

### 2.1 Ethical standards

This study was approved by the Ethics Committees of The First Affiliated Hospital of University of South China and conducted according to the Helsinki Declaration. Informed consents were obtained from all patients. Animal study was approved by the Institutional Animal Care and Use Committee (IACUC) of The First Affiliated Hospital of University of South China and conducted according to IACUC protocol.

### 2.2 Cell culture

Cell lines were bought from ATCC (USA) and passaged within six months. Cells were cultured according to the manufacturer's instruction and re‐authenticated by short tandem repeat DNA profiling before used.

### 2.3 Quantitative real‐time polymerase chain reaction

Total RNA was isolated by TRIzol (Invitrogen, USA). Cytoplasmic and nuclear RNA was isolated by PARIS™ Kit (Invitrogen). cDNA was synthesized by PrimeScript RT reagent kit (Takara, China), and RT‐PCR was performed with SYBR Premix Ex Taq (Takara) and CFX96 Real‐time PCR system (Bio‐Rad, USA). The relative fold‐change was calculated by the 2^−ΔΔCt^ method. The primers were synthesized by Invitrogen (Table S1).

### 2.4 Cell counting kit‐8 (CCK‐8) assay

Cells (1 × 10^3^) were seeded 24 hours before transfection. Forty‐eight hours after transfection, CCK‐8 solution (10 μL) was added. Two hours later, the absorbance at 490 nmol/L was measured by Spectra Max 250 spectrophotometer (Molecular Devices, USA).

### 2.5 Apoptosis assay

The cells were transfected as described, and the apoptotic rate was detected. Annexin V/propidium iodide staining and flow cytometry were performed with Annexin V‐fluorescein isothiocynate Apoptosis Detection Kit (KeyGen, Nanjing, China).

### 2.6 Transwell assay

Cells were seeded onto the EC matrix (Millipore, Germany) and 20% foetal bovine serum was added to the lower chamber. Forty‐eight hours later, non‐invading cells were removed while invading cells were fixed and stained, imaged and counted.

### 2.7 Mouse xenograft assay

Four‐week‐old BALB/c nude mice were subcutaneously injected with 2 × 10^6^ OV119 cells (five mice per group to provide a power of 90% for a significance level of 0.05 with a two‐tailed *t* test) and treated with intratumoural injection (40 μL si‐NC or si‐circEPSTI1).

For metastasis assays, OV119 cells were inoculated through tail vein into nude mice (five mice per group to provide a power of 90% for a significance level of 0.05 with a two‐tailed *t* test). Four weeks later, mice were sacrificed and lung metastatic nodules were counted.

### 2.8 Luciferase assay

Luciferase reporter vector with 3′‐UTR of EPSTI1 or circEPSTI1 were constructed. Cells were co‐transfected with luciferase reporter vectors and miR‐942 mimics by Lipofectamine 2000 (Invitrogen). Forty‐eight hours after incubation, the luciferase activities were quantified by dual‐luciferase reporter assay (Promega, USA).

### 2.9 RNA immunoprecipitation assay

Cells were co‐transfected with MS2bs‐circEPSTI1, MS2bs‐circEPSTI1mt or control MS2bs‐Rluc by Lipofectamine 2000. Forty‐eight hours later RIP was performed with Magna RIP RNA‐Binding Protein Immunoprecipitation Kit (Millipore). For RIP assay on Ago2, 48 hours after transfection, RIP was performed with an anti‐Ago2 antibody (Millipore).

### 2.10 Western blot analysis

Proteins were separated by 10% SDS‐PAGE and transferred to PVDF membrane (Millipore) and incubated with 5% skim milk before incubated with antibody against EPSTI1 (1:500, Santa cruz, USA). Then the membrane was incubated with secondary antibody (1:3000, CST, USA) and detected by chemiluminescence. Anti‐β‐actin antibody (1:1000, Affinity, USA) was used as control.

### 2.11 Statistical analysis

Statistical analysis was performed by SPSS 19.0 software. Comparisons between groups were performed by *t* tests and *χ* ^2^ tests. Unless otherwise indicated, data are presented as the mean ± SEM of triplicate independent experiments. *P* < 0.05 was considered statistically significant.

## 3 RESULTS

### 3.1 circEPSTI1 is up‐regulated and promotes ovarian cancer progression in vitro

**Figure 1:**
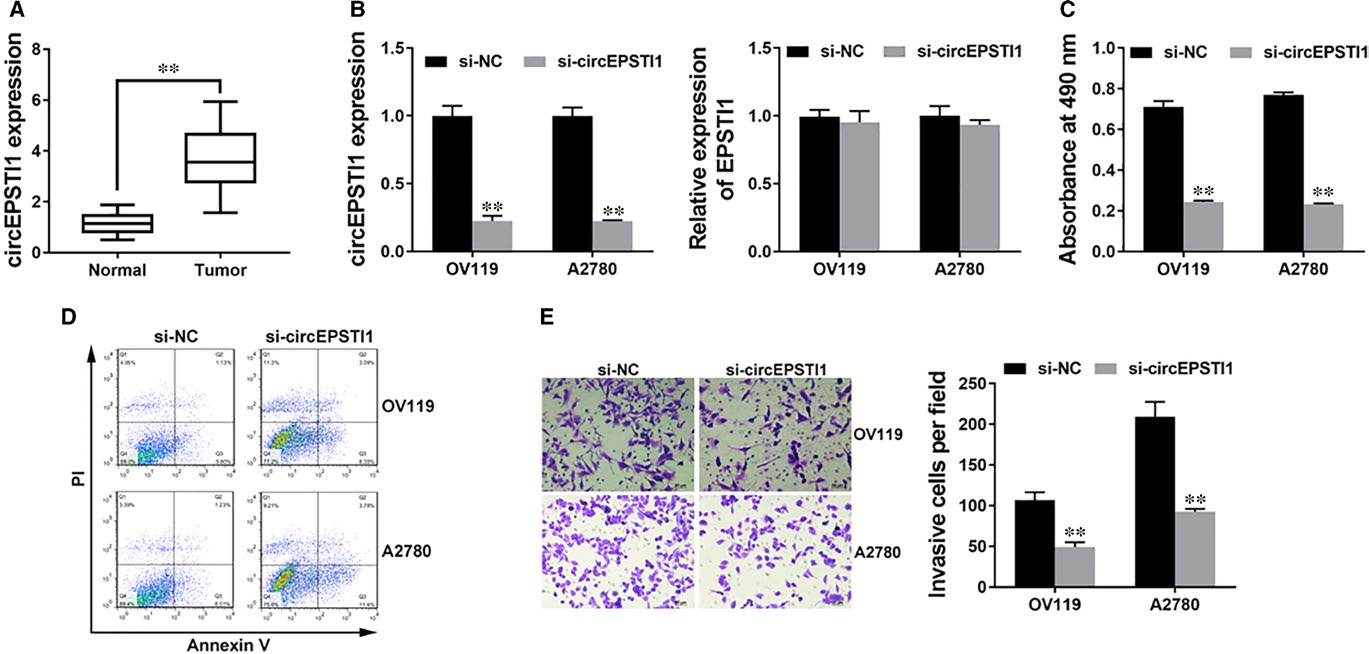
circEPSTI1 is up‐regulated and promotes ovarian cancer progression in vitro A. The expression of circEPSTI1 in 50 paired ovarian cancer tissue (Tumour) and adjacent normal tissues (Normal). B, Cells were transfected with si‐NC or si‐circEPSTI1, and the expression of circEPSTI1 (left) and EPSTI1 (right) were determined by qRT‐PCR analysis. C, CCK‐8 assay was performed to assess cell growth. D, Cell apoptosis was determined 48 h after transfection. E, Transwell assay was performed. Representative images of invaded cells are shown in the left panel, and the results are summarized in the right panel. Original magnification, ×200. **P < 0.01

To explore the expression of circEPSTI1 in ovarian cancer, qRT‐PCR was performed in 50 paired ovarian cancer tissue (Tumour) and adjacent normal tissues (Normal) and we found circEPSTI1 overexpressed in ovarian cancer tissues (Figure 1A).

Since circEPSTI1 was up‐regulated, we knocked down circEPSTI1 to explore its function in ovarian cancer progression. Figure 1B showed that the inhibition of circEPSTI1 was successful. And we confirmed that si‐circEPSTI1 can only knockdown the circular transcript without prejudice to the linear form EPSTI1 (Figure 1B). CCK‐8 assay showed that inhibition of circEPSTI1 inhibited cell proliferation (Figure 1C). Apoptosis assay revealed that inhibition of circEPSTI1 led to increased apoptosis (Figure 1D). Transwell assay showed that inhibition of circEPSTI1 suppressed cell invasion (Figure 1E). All these findings indicate that inhibition of circEPSTI1 suppresses cell proliferation and invasion, but induces cell apoptosis in ovarian cancer in vitro.

### 3.2 circEPSTI1 promotes ovarian cancer progression in vivo

**Figure 2:**
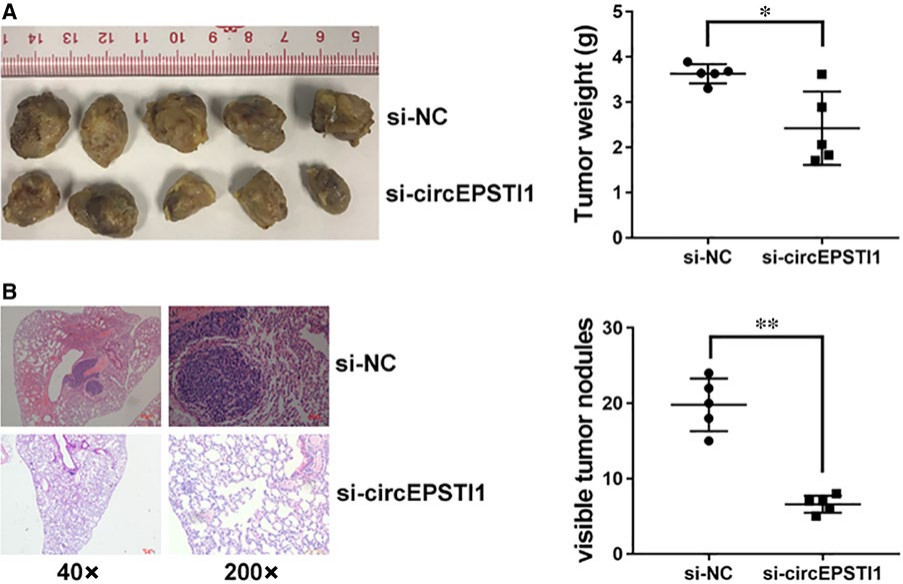
circEPSTI1 promotes ovarian cancer progression in vivo A. Representative images of xenografts tumour in nude mice are shown in the left panel. The weights of xenograft tumours are summarized in the right panel. B, Representative images of HE stained lung metastatic nodules are shown in the left panel. The number of metastatic nodules was summarized in the right panel. *P < 0.05, **P < 0.01

To further explore the function of circEPSTI1 in vivo, we performed mouse xenograft assays. And knockdown of circEPSTI1 resulted in decreased tumour growth (Figure 2A). Moreover, lung metastatic nodules decreased after inhibition of circEPSTI1 (Figure 2B). All these findings indicate that inhibition of circEPSTI1 suppresses cell proliferation and invasion in ovarian cancer in vivo.

### 3.3 circEPSTI1 acts as a decoy for miR‐942

**Figure 3:**
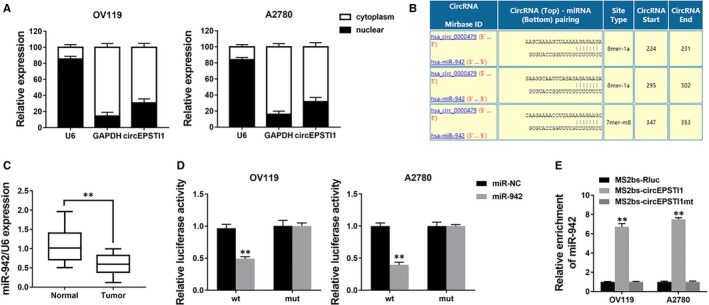
circEPSTI1 acts as a decoy for miR‐942 A. The levels of U6, GAPDH and circEPSTI1 were assessed by qRT‐PCR in nuclear and cytoplasmic fractions. B, The predicted binding sites of miR‐942 within circEPSTI1 were shown. C, The expression of miR‐942 in 50 paired ovarian cancer tissue (Tumour) and adjacent normal tissues (Normal). D, Luciferase reporter assay of cells co‐transfected with miR‐942 mimics and luciferase reporter containing circEPSTI1 (wt) or mutant construct (mut). E, MS2bp‐MS2bs based RIP assay in cells transfected with MS2bs‐circEPSTI1, MS2bs‐circEPSTI1mt or MS2bs‐Rluc. **P < 0.01

We explored the intracellular location of circEPSTI1 and found that circEPSTI1 mostly distributed in the cytoplasm (Figure 3A), suggesting that circEPSTI1 might act as a miRNA sponge to decoy miRNAs. Therefore, we investigated the potential circRNA/miRNA interaction by Circular RNA Interactome (https://circinteractome.nia.nih.gov/index.html) and found complementary sites of miR‐942 within circEPSTI1 sequence (Figure 3B). Moreover, the expression of miR‐942 was down‐regulated in ovarian cancer tissues (Figure 3C). Next, luciferase assays was performed to explore whether miR‐942 could bind to circEPSTI1. And the luciferase intensity decreased when co‐transfected with luciferase reporters and miR‐942 mimics (Figure 3D). To confirm the direct binding of circEPSTI1 and miR‐942, MS2bp‐MS2bs based RIP assay was performed. We found that miR‐942 was mostly enriched in MS2bs‐circEPSTI1 group, indicating the specific interaction of circEPSTI1 and miR‐942 (Figure 3E). Together, our data confirmed that circEPSTI1 could interact with miR‐942 and act as a decoy for miR‐942.

### 3.4 circEPSTI1 regulates EPSTI1 via miR‐942

**Figure 4:**
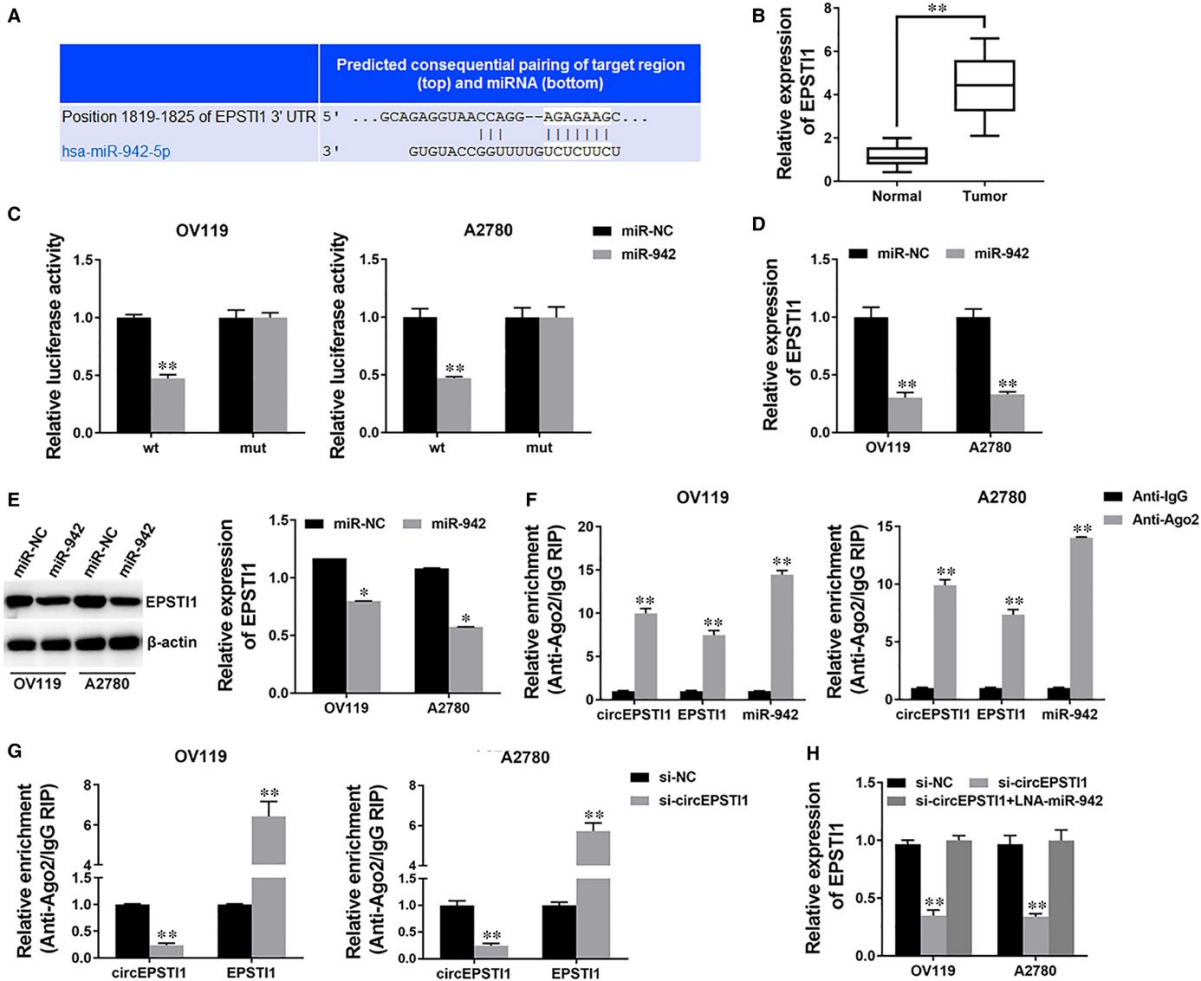
circEPSTI1 regulates EPSTI1 via miR‐942 A. The predicted binding sites of miR‐942 within EPSTI1 3’UTR were shown. B, The expression of EPSTI1 in 50 paired ovarian cancer tissue (Tumour) and adjacent normal tissues (Normal). C, Luciferase reporter assay of cells co‐transfected with miR‐942 mimics and luciferase reporter containing EPSTI1 3′UTR (wt) or mutant construct (mut). D, Cells were transfected as described, and the expression of EPSTI1 was determined by qRT‐PCR. E, Cells were transfected as described, and the expression of EPSTI1 was determined by Western blot (left) and quantified (right). F, RIP assay showing the enrichment of circEPSTI1, EPSTI1 and miR‐942 on Ago2 relative to IgG. G, Cells were transfected as described and RIP assay on Ago2 was performed. H, Cells were transfected as described, and the expression of EPSTI1 was determined by qRT‐PCR. **P < 0.01

To investigate whether circEPSTI1 decoyed miR‐942 to regulate the expression of target genes, we used TargetScan to identify the target genes of miR‐942 and EPSTI1 was predicted (Figure 4A). We detected the expression of EPSTI1 and found that EPSTI1 was also overexpressed in ovarian cancer tissues (Figure 4B). Next, we performed luciferase reporter assay and the luciferase activity significantly decreased when co‐transfected with miR‐942 mimics and luciferase reporter (Figure 4C). And the expression of EPSTI1 was inhibited by miR‐942 (Figure 4D,E), indicating that EPSTI1 was a direct target of miR‐942.

Next, a RIP assay on Ago2 showed that circEPSTI1, EPSTI1 and miR‐942 were mainly enriched to Ago2 (Figure 4F), indicating that circEPSTI1 and EPSTI1 are recruited to an Ago2‐related RISC where they interact with miR‐942. In addition, inhibition of circEPSTI1 decreased the enrichment of Ago2 to circEPSTI1, but increased the enrichment of Ago2 to EPSTI1 (Figure 4G), indicating that circEPSTI1 could function as a ceRNA and compete with EPSTI1 for binding miRNAs. Therefore, we explored the expression of EPSTI1 after circEPSTI1 knockdown and found it decreased, but inhibition of miR‐942 could reverse the repression (Figure 4H). These results indicate that circEPSTI1 could serve as a ceRNA to regulate EPSTI1 expression via decoying miR‐942.

## 4 DISCUSSION

Increasing studies have reported that circRNAs play vital roles in cancer.^9^ In gastric cancer, circ‐KIAA1244 is correlated with cancer progression and patients’ outcome.^10^ In non‑small cell lung cancer, hsa_circ_0033155 acts as a tumour suppressor to regulate cell proliferation, colony formation and migration.^11^ However, there are only a few reports exploring circRNAs in ovarian cancer. Here, we detected the expression of circEPSTI1 and found circEPSTI1 up‐regulated in ovarian cancer. Subsequent experiments revealed that inhibition of circEPSTI1 suppressed cell proliferation and invasion, but induced cell apoptosis, indicating that circEPSTI1 could regulate ovarian cancer progression. Therefore, circEPSTI1 could be a potential biomarker and therapeutic target.

CircRNAs have been reported to sponge miRNA and regulate gene expression.^12^ In malignant melanoma, hsa_circ_0025039 promotes cell growth, invasion and glucose metabolism via sponging miR‐198.^13^ In colon cancer, circRNA‐ACAP2 sponges miR‐21‐5p to regulate cell proliferation, migration and invasion.^14^ In ovarian cancer, hsa_circ_0061140 affects cell growth and metastasis through sponging miR‐370.^15^ Here, we found that circEPSTI1 could bind to miR‐942 and acted as a miRNA sponge.

miR‐942 is involved in cancer progression. Forced expression of miR‐942 significantly changes the drug sensitive cells to resistant in hepatocellular carcinoma and gastric cancer.^16^ In esophageal squamous cell carcinoma, miR‐942 promotes cancer stem cell‐like traits through activation of Wnt/β‐catenin signalling pathway.^17^ In colorectal cancer, long non‐coding RNA linc00675 suppresses cell proliferation and metastasis via regulating miR‐942.^18^ Here, we found that miR‐942 was down‐regulated in ovarian cancer.

In addition, we found that EPSTI1 was a target gene of miR‐942. EPSTI1 has been reported overexpressed in multiple cancers and involved in cancer progression. In pancreatic cancer, EPSTI1 is associated with resistance to oncolytic vesicular stomatitis virus.^19^ In breast cancer, abnormal expression of EPSTI1 is associated with cell invasion, metastasis^20^ and apoptosis.^21^ Here, we found that EPSTI1 was overexpressed in ovarian cancer. Subsequent experiments suggest that circEPSTI1 acted as a decoy for miR‐942 to regulate EPSTI1 expression. Inhibition of circEPSTI1 reduced EPSTI1 expression and inhibition of miR‐942 could reverse it.

In summary, we found that circEPSTI1 was overexpressed in ovarian cancer. Inhibition of circEPSTI1 suppressed cell proliferation and invasion, but induced cell apoptosis. circEPSTI1 regulated EPSTI1 expression via sponging miR‐942. circEPSTI1 could be a potential biomarker and therapeutic target in ovarian cancer.

## CONFLICT OF INTEREST

The authors declare no conflict of interest.
